# Trigeminal Function in Sino-Nasal Health and Disease

**DOI:** 10.3390/biomedicines11071778

**Published:** 2023-06-21

**Authors:** Dennis Shusterman

**Affiliations:** Division of Occupational, Environmental and Climate Medicine, University of California, San Francisco, CA 94143-0843, USA; dennis.shusterman@ucsf.edu

**Keywords:** nose, paranasal sinuses, larynx, rhinitis, sinusitis, cough, trigeminal nerve, glossopharyngeal nerve, stimulation, receptors, reflexes, irritation, airflow, meninges, headache

## Abstract

The upper airway (nasal passages, paranasal sinuses, pharynx, and glottis) provides the sentinel portion of the human respiratory tract, with the combined senses of olfaction (cranial nerve I) and trigeminal sensation (cranial nerve V) signaling the quality of inspired air. Trigeminal function also complements the sense of taste (in turn mediated by cranial nerves VII, IX and X), and participates in the genesis of taste aversions. The ability of trigeminal stimulation in the upper aero-digestive tract to trigger a variety of respiratory and behavioral reflexes has long been recognized. In this context, the last three decades has seen a proliferation of observations at a molecular level regarding the mechanisms of olfaction, irritation, and gustation. Concurrently, an ever-widening network of physiological interactions between olfaction, taste, and trigeminal function has been uncovered. The objective of this review is to summarize the relatively recent expansion of research in this sub-field of sensory science, and to explore the clinical and therapeutic implications thereof.

## 1. Introduction

**Figure 1:**
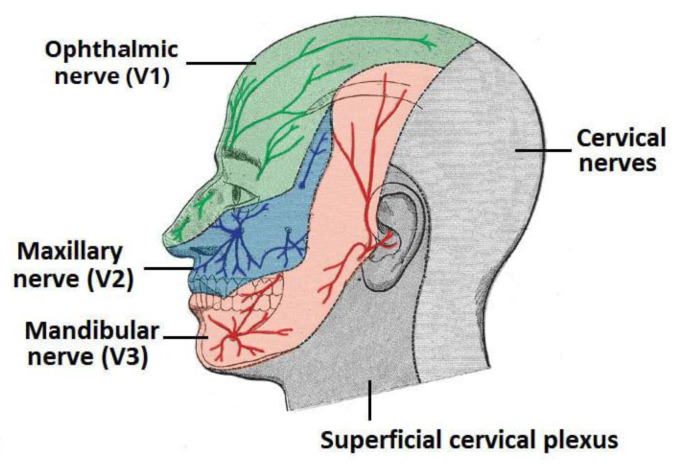
Surface innervation by the fifth (trigeminal) cranial nerve. From reference [1].

The face and anterior scalp, along with the mucous membranes of the eyes, nose, paranasal sinuses, and oral cavity, obtain their principal somatosensory innervation via the trigeminal nerve (CrN V)—Figure 1. Trigeminal sensation is supplemented, in turn, by the glossopharyngeal nerve (CrN IX) in the pharynx, and by the vagus nerve (CrN X) in the larynx. Complementing somatosensation are the special senses of olfaction and taste, with the olfactory nerve (CrN I) innervating the superior portion of the nasal cavity, and canonical taste receptors being expressed on facial, glossopharyngeal, and vagus nerve branches (CrN VII, IX and X). Together, these afferents respond to mechanical, thermal, and chemical stimuli, the interpretation of which is essential for protective, respiratory, and ingestive behaviors.

Chemical nociception, defined as the perception of noxious chemical stimuli, has an established research history, dating back at least to Sherrington’s initial use of the word “nociceptor” in 1906 [2,3]. More recently employed terms have included “the common chemical sense”, “chemalgia”, “sensory irritation”, and “chemesthesis” [4,5,6,7]. The most recently coined term—chemesthesis (literally, “chemical feel”)—alludes to the fact that, at least in the context of the upper aerodigestive tract (and independent of taste and smell), chemicals can impart a variety of sensations, including astringency, cooling, warming, burning, itching, or pain. These sensations exist on a spectrum, which—while clearly encompassing danger signals—also includes sought-after qualities of foods and beverages (such as the “burn” of chili peppers or the tingling of carbonated drinks). To the extent that we consider oro-nasal and pharyngeal trigeminal stimulation qualitatively, then, we must consider not only normal physiological and protective (“chemofensor”) reflexes, but also ingestive aesthetics [8,9].

Building on a foundation of psychophysics, electrophysiology, and pharmacology, the study of chemesthesis has effectively matured with the identification and cloning of specific irritant receptors. Molecular biologic tools were first applied to the study of nicotinic and purinergic receptors starting in the early 1980s, and by the late 1990s were also used to characterize the nociceptive receptor for hydrogen ions (H^+^) and capsaicin (the active principal in chili peppers) [10,11]. The capsaicin receptor also responds to both heat and acid pH, and it was established that these three stimuli potentiated the response of one another [12]. Although initially termed “vanilloid receptor 1” (VR1), this was the first of several ligand-gated cation channels identified in vertebrates that structurally resembled the family of “transient receptor potential” (TRP) receptors first cloned in drosophila [13]. Given this structural homology, VR1 was destined to be re-named “TRPV1” (“V” for vanilloid), and over the ensuing decade, two more explicitly chemesthetic ion channels would also be cloned. These included TRPM8 (“M” signifying “melastatin”)—responding to menthol and cold temperatures, and TRPA1 (“A” signifying ankyrin)—responding to a wide variety of natural and anthropogenic irritant chemicals and to extreme cold temperatures [14,15,16].

These achievements capped a three-decade period (~1991–2021) during which the work of sensory scientists garnered two Nobel Prizes in Medicine or Physiology: Linda Buck and Richard Axel in 2004 (for unraveling the molecular mechanism of olfactory transduction), and David Julius and Arden Patapoutian in 2021 (for elucidating transduction mechanisms in chemesthesis and tactile touch) [17,18]. In each case, these achievements reflected the culmination of years of behavioral, psychophysical, pharmacologic, electrophysiologic, and molecular biologic research in multiple laboratories worldwide, as exemplified by the case of TRPM8 [19].

In the pages that follow, we will review the range of stimuli—both natural and anthropogenic—that are detected by the trigeminal nerve (in concert with the senses of smell and taste) and will explore the wide variety of physiologic and pathophysiologic reflexes in which they participate. In this process, we will allude to a broad spectrum of functional and aesthetic functions attributable to this important neurologic structure.

## 2. Anatomy and Physiology

### 2.1. Anatomy

**Figure 2:**
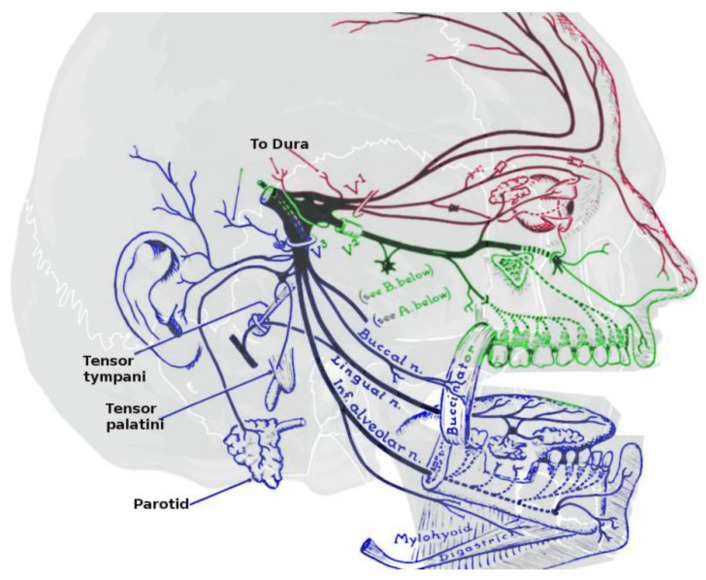
Subsurface distribution of the trigeminal nerve (CrN V) in humans. Branches: Ophthalmic (V1 red), maxillary (V2 green), and mandibular (V3 blue). From reference [1].

The trigeminal nerve is the largest (and arguably, one of the more complex) of the twelve cranial nerves. Its neurons are classified as “pseudo-unipolar” (with a single axon branching both centrally and distally from their cell bodies), and hence are capable of both afferent and efferent conduction (of relevance when considering the “axon reflex”). Nevertheless, the vast majority are sensory in nature (with the exclusion of mandibular branch neurons innervating the muscles of mastication). CrN V’s cell bodies reside in the trigeminal ganglion (also referred to as the “semilunar ganglion” [for its shape] or “Gasserian ganglion” [for the anatomists who first described it in the 18th century]). The CNS nuclei to which they synapse range in location from the mid-brain rostrally to the medulla oblongata caudally. The peripheral ramifications of CrN V form three branches: V1 (ophthalmic), V2 (maxillary), and V3 (mandibular). Each of these trunks, in turn, divide into between 3 and 14 individual nerves (Figure 2).

**Figure 3:**
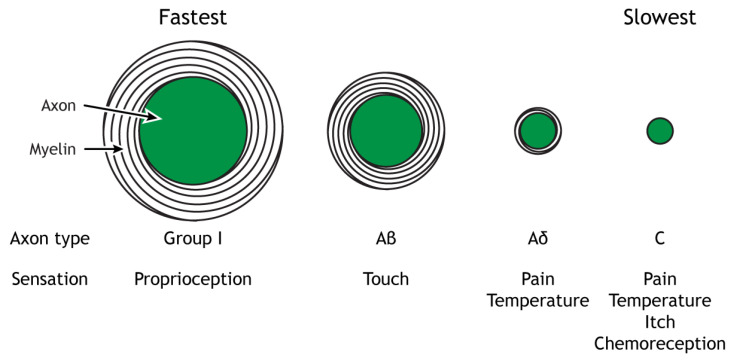
Nerve fiber sub-populations, arranged from largest diameter and myelinated (fastest) to smallest diameter/unmyelinated (slowest). From reference [20].

Histologically, the sensory fibers of CrN V include large-diameter myelinated Aα (“Group I”) fibers (conveying proprioception), Aβ fibers (light touch and vibration), small-diameter myelinated Aδ fibers (sharp pain and cold), and small, unmyelinated C fibers (dull pain and heat)—Figure 3. Conduction speed in these fibers is influenced by characteristics such as axon diameter and degree of myelination (with greater diameter and more thoroughly myelinated fibers conducting impulses more rapidly).

**Figure 4:**
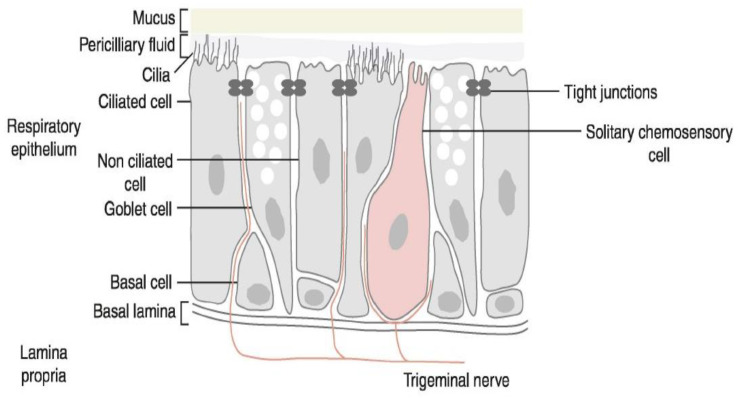
Mucosal “free” nerve endings (terminating below “tight junctions”) and solitary chemosensory cells (in contact with the mucosal lumen). With permission from reference [21].

With the exception of specialized tactile nerve functions, the preponderance of trigeminal nerve endings, while liberally endowed with nociceptive ion channels, are unadorned by specialized sensory organs (hence the appellation, “free nerve endings”). Importantly, we now know that so-called “solitary chemoreceptor cells” (SCCs)—initially described in fish—are not only present in humans but are in direct contact with the mucosal lumen and can transduce a variety of intraluminal chemical signals (secondarily stimulating adjacent trigeminal nerve endings—Figure 4).

It has been suggested that the encoding of chemesthetic qualities begins with signal transduction in different subsets of afferent neurons endowed with specific nociceptive ion channels (i.e., a labeled line model) [22,23]. As the heterogeneity of nociceptive ion channels is documented in additional afferent fiber sub-populations, this model is appearing progressively less viable (i.e., competing vs. pattern, intensity, and gated control models) [24].

### 2.2. Mucosal Chemoreceptors

#### 2.2.1. Classes of Receptors

**Figure 5:**
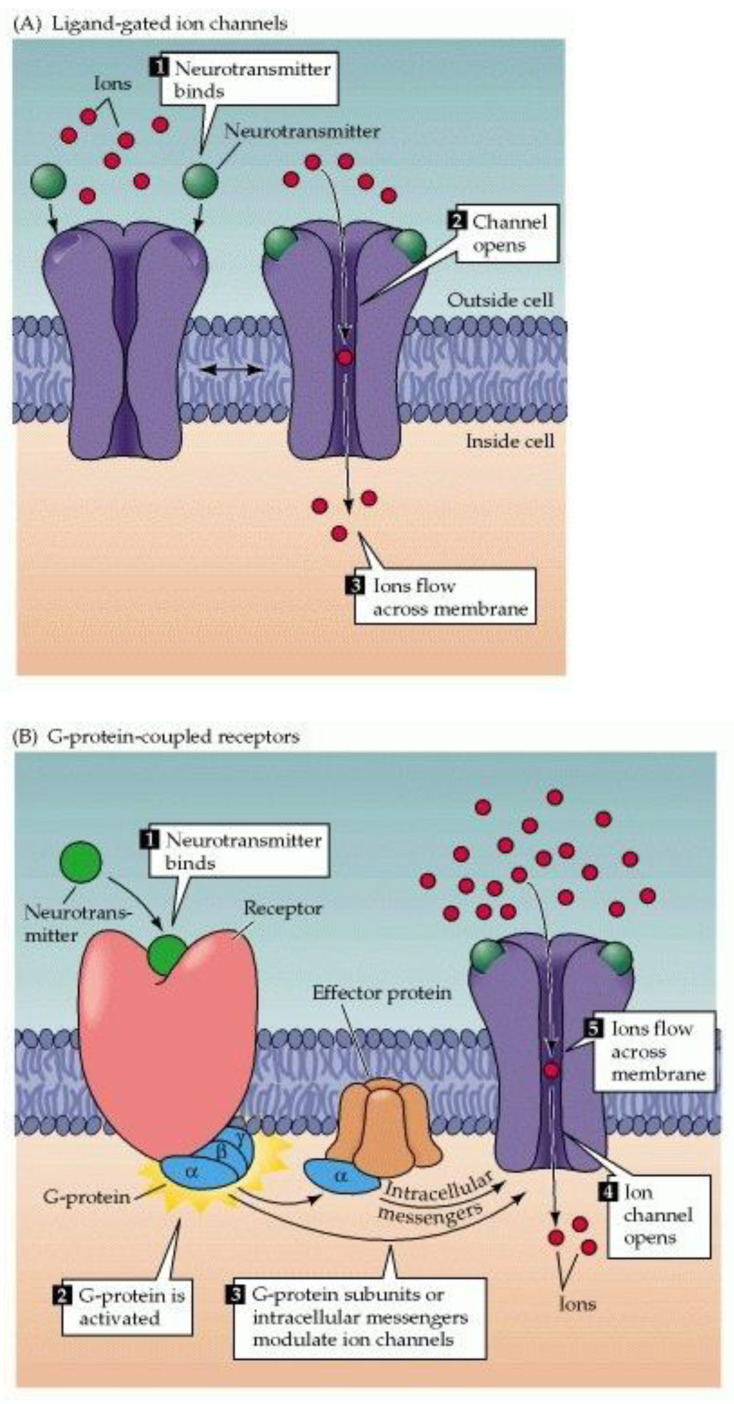
Two major classes of membrane receptors: (**A**) ion channels and (**B**) GPCRs. Most nociceptors (e.g., TRPV1, TRPM8, TRPA1, ASIC, nAChR, and P2X) are ion channels. With permission from reference [25].

Membrane receptors gating the entry of cations into cells are generally classified as either “ionotropic” (ion channels responding directly to ligands) or “metabotropic” (G-protein-coupled receptors or “GPCRs”, acting through “second messengers”)—Figure 5. Since ion channels can, in general, respond to stimuli more quickly than GPCRs, it is no surprise that they have come to predominate in sensory systems for which reaction time is critical (such as touch, hearing, and chemesthesis). Examples of ionotropic nociceptors include TRP channels, acid-sensitive ion channels (ASICs), nicotinic acetylcholine (Ach) receptors, and purinergic (P2X) receptors (responding to extracellular adenosine triphosphate [ATP]).

Nociceptors are found on both excitable and non-excitable cells. In this context, the distinction between “physiologic” and “nociceptive” functions can be somewhat blurred. For example, ATP, while having a physiologic role in an intracellular setting, can signal cell damage if elevated extracellularly, with extracellularly exposed P2X receptors sending signals that the CNS interprets as “painful” [26]. Similarly, while sensing minor variations in pH is important for respiratory regulation, the presence of significant localized extracellular acidosis—as can occur with ischemia or infection—may transduce pain signals via ASICs [27].

Given the infusion of new pharmacologic data involving nociceptive ion channels over the last few decades, some selectivity is necessary in a brief review such as this. Accordingly, we will concentrate on: (1) the role that nociceptors (specifically, various TRP channels) play in temperature-sensing; and (2) the relative specificity with which various ligands (both natural and anthropogenic) interact with nociceptive ion channels. Not covered in detail are: endogenous biochemicals acting as receptor ligands or modulating agents; the general topic of nociceptor antagonists; or the interaction of trigeminal and olfactory function. For these topics, the reader is directed to in-depth reviews [28,29,30].

#### 2.2.2. Temperature Sensing

**Figure 6:**
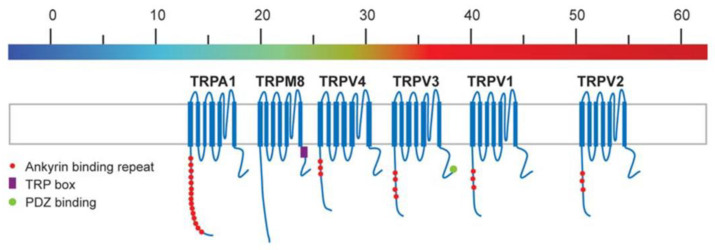
Thermal response spectra of common human TRP (‘thermoTRP’) channels. Adapted from reference [31].

As depicted in Figure 6, different TRP channels activate at different points in the temperature spectrum. At the low extreme is TRPA1, the activity of which begins to increase as the temperature dips below 17 °C (i.e., “noxious cold”.). Just above TRPA1 in the continuum is TRPM8 (sometimes referred to as the “menthol/cool receptor”), which activates below 25 °C. Interposed between TRPM8 and TRPV1 are TRPV4 and TRPV3, the latter of which activates between 32 and 39 °C. TRPV1, the capsaicin receptor, activates above 42–43 °C (temperatures most individuals would find at least borderline uncomfortable), and TRPV2—at the upper end of the spectrum—activates above 52 °C (temperatures at which tissue damage occurs quite rapidly). The collective responsiveness of this array of TRP channels effectively comprises a biomolecular thermometer [31].

#### 2.2.3. Ligand-Receptor Interactions

In his 2021 Nobel Prize acceptance speech, David Julius lauded the historical body of drug research using natural products by referring to “natural products and folk medicine” as “pharmacology honed by evolution” [32]. In his (and others’) laboratories, three natural products—capsaicin, menthol, and mustard oil (allyl isothiocyanate)—were keys to characterizing major nociceptors (i.e., TRPV1, TRPM8, and TRPA1, respectively). As if to emphasize the point, both insect venoms and plant defensins also played a supporting role in this process. Accordingly, we would be remiss were we not to give equal weight to both natural products and anthropogenic chemicals in terms of ligand–receptor interactions. Below, we consider selected [TRP family] ion-channel nociceptors in their order of molecular cloning: TRPV1, followed by TRPM8, and finally TRPA1 (Table 1, Table 2, Table 3, Table 4, Table 5 and Table 6). Other identified TRP ion channels whose functions are not explicitly sensory (e.g., TRP 3, 4 and 5) are reviewed in detail elsewhere [33,34].

**TRPV1**

**Table 1:** TRPV1 Ligands (Naturally occurring).

Capsaicin		Resineriferatoxin
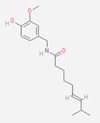	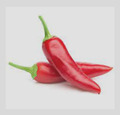	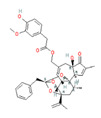
Chili peppers (*Capsicum annuum var*)		Resin spurge (*Eurphorbia resinifera*)
Allicin	Camphorin	Piperine
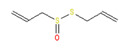	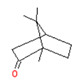	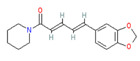
Garlic (*Allium sativum*)	Camphor laurel (*Cinnamonum camphera*)	Black pepper (*Piper nigrum*)

**Capsaicin** (8-methyl-N-vanillyl-6-nonenamide) has been considered by many as the prototype irritant, leading off a series of human TRP channel isolations and cloning procedures culminating between 1997 and 2004. Capsaicin-containing chili peppers have been known from time immemorial as a source of pungency in food and as a potential food preservative. Psychophysically, the capsaicin concentration in chili peppers has been quantified using the Scoville scale, with bell peppers rated at 0, jalapenos at ~5000, habaneros at ~100,000, “ghost” peppers at ~1,000,000, and pure capsaicin at 16,000,000 [35]. Resiniferitoxin, a substance derived from a cactus-like plant, produces stronger and more sustained cellular depolarization than pure capsaicin, with an estimated Scoville rating of 16,000,000,000. As a consequence, it is utilized exclusively in laboratory experiments.

In a broader biological context, TRPV1 receptors vary between species, being fully functional in mammals, but in birds reactive to heat and acid, but not to capsaicin. This biochemical oddity renders birds capable of ingesting chili seeds (and subsequently spreading them geographically). While not highly volatile, minute quantities of aerosolized capsaicin can irritate mucous membranes (inducing eye irritation and cough).

Capsaicin and related vanilloids are lipophilic and are capable of being absorbed through intact cornified skin (although not as rapidly or completely as through mucous membranes). Capsaicin’s lipophilicity does, however, enable it to cross cell membranes, giving it access to TRPV1′s active capsaicin binding site (which is, interestingly, intracellular in location). Allicin, camphorin, and piperine are other naturally occurring TRPV1 ligands that produce a “stinging” or “burning” sensation in human mucous membranes, although not to the same extent as capsaicin (Table 1, above).

**Table 2:** TRPV1 Ligands (Anthropogenic/Industrial).

Nonanoyl-Vanillamide	Olvanil	Glyceryl Nonivamide
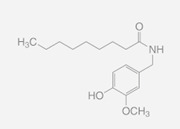	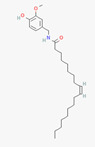	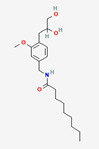

Synthetically produced compounds such as nonanoyl-vanillamide (N-[(4-hydroxy-3-methoxyphenyl)methyl]nonanamide; Table 2, above) can approximate the biological activity of natural capsaicin, and in fact may occur naturally in very low concentrations. Chemists have also experimented with fully synthetic vanilloid structures to achieve desirable pharmacologic endpoints (e.g., desensitization of nociceptive C-fibers) while minimizing undesirable effects (e.g., stinging and burning). Two products of this line of experimentation have been olvanil ((Z)-N-[(4-hydroxy-3-methoxyphenyl)methyl]octadec-9-enamide) and glyceryl nonivamide (N-[[4-(2,3-dihydroxypropyl)-3-methoxyphenyl]methyl]nonanamide). The future synthesis of additional synthetic vanilloids is likely, given the level of interest in this class of agents for pain management [36,37].

**TRPM8**

**Table 3:** TRPM8 Ligands (*Naturally occurring*).

(-)-Menthol		Eucalyptol/Cineole
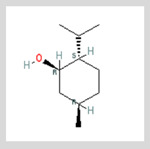	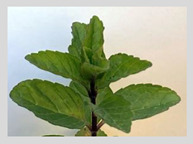	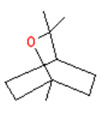
Spearmint (*Mentha spicata*)		
Geraniol		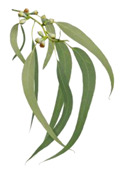
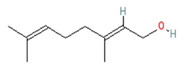	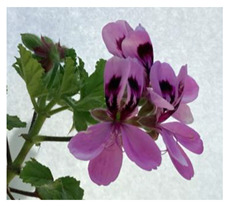	
Rose/Citronella/Geranium (*Oils*)		Blue Gum (*Eucalyptus globulus*)

**Menthol** (5-methyl-2-propan-2-ylcyclohexan-1-ol) is most often extracted directly from mint plants (*Menta* spp.). It has a distinctive odor, along with a chemesthetic impact most often described as “cooling” and/or “numbing”. The menthol molecule is optically active around three centers (yielding eight stereoisomers), but only one of these—“(-)-menthol” (or “L-menthol”)—fully expresses the sensory characteristics normally associated with the compound [38]. (-)-Menthol is soluble in alcohols and other non-polar organics, is sparing soluble in water, and has moderately high volatility. Other naturally occurring TRPM8 agonists include eucalyptol, cineol, and various plant oils (rose, citronella and geranium, among others (Table 3, above).

Given the compound’s volatility, menthol-containing formulations can either be applied topically near the breathing zone of patients (to deliver its vapors to the nose and upper airway), or as an ingredient in lozenges or nasal sprays. They are distributed as over-the-counter remedies to be employed as “decongestants” for individuals suffering a sense of nasal airflow obstruction from viral upper respiratory infections or allergies, and also as a cough suppressant. Interestingly, while the application of menthol tends to produce improvement in patients’ subjective rating of nasal patency (i.e., perception of nasal “openness”), it is not accompanied by concomitant changes in objective measures thereof, leading to speculation that subjective patency is mediated by a sensation of nasal mucosal cooling rather than by aerodynamic forces per se. Evidence regarding this hypothesis is reviewed in Section 2.3.4, below.

In addition to providing subjective relief from nasal congestion, menthol has been used to “mask” the undesirable effects of other agents. For example, nicotine imparts a slight irritancy (“bite”) to cigarette smoke, while at the same time triggering psychopharmacologic effects in the central nervous system that habitual users find rewarding [39]. However, a significant subset of smokers describes cigarette smoke’s irritancy as “harshness”. Tobacco purveyors have learned that, for smokers who find airway irritation objectionable, the addition of menthol can perceptually blunt the irritancy of nicotine without affecting the psychopharmacologic effects that the smokers desire [40].

**Table 4:** TRPM8 Ligands (Anthropogenic/Industrial).

Icilin	Cubebol	WS-12
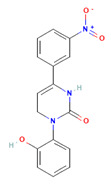	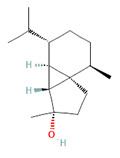	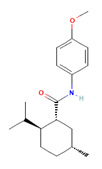

Given the range of potential impacts of menthol-like medications, extensive energy has been invested in selectively recreating its effects with synthetic chemicals (Table 4, above). For example, several synthetic TRPM8 ligands have been synthesized, including icillin [3-(2-hydroxyphenyl)-6-(3-nitrophenyl)-1,4-dihydropyrimidin-2-one], cubebol [(1R,4S,5R,6R,7S, 10R)- 4,10-dimethyl-7-propan-2-yltricyclo [4.4.0.0^1,5^]decan-4-ol], and WS-12 [(1R,2S,5R)-N-(4-methoxyphenyl)-5-methyl-2-propan-2-ylcyclohexane-1-carboxamide] (Table 4). Explicit benefits of developing TRPM8 agonists with selective effects include treating symptoms associated with dry eye and burning mouth syndromes, as well as alleviating itching due to dry skin [41]. Less widely discussed has been the potential use of non-odorous cooling agents to circumvent the impact of tobacco-control legislation regulating the addition of flavoring agents (including menthol) to cigarettes, smokeless tobacco, and electronic nicotine-delivery systems [42].

**TRPA1**

**Allyl isothiocyanate**—found in horseradish, mustard oil, and other pungent Brassica plants—has been treated as the prototype ligand for TRPA1. Initially labeled *ANKTM1*, TRPA1 acts not only as a ligand-gated ion channel, but also as a thermoreceptor, responding to “noxious cold” (<17 °C, as noted above). Although TRPA1 is the most recently cloned of the three nociceptive TRP channels reviewed here, it responds to the broadest array of both naturally occurring and anthropogenic/industrial agents. The wide range of botanical sources for TRPA1 ligands is apparent in Table 5 (below) and includes substantial overlap with TRPV1 ligands. Of at least equal importance to environmental scientists, it has been established that TRPA1 activation occurs from an extraordinarily wide range of synthetic and industrial chemicals. Prominent among these are acetaldehyde, acrolein, ammonium chloride, chloropicrin, chlorine, cigarette smoke, formalin, hydrogen peroxide, hypochlorite salts, methyl isocyanate (MIC), methyl isothiocyanate (MITC), ozone, and toluene diisocyanate (TDI), among others. Of importance, TRPA1 is activated by both reversible ligand binding and by covalent (electrophilic) reactions with air pollutants [43].

**Table 5:** TRPA1 Ligands/Reactants (Naturally occurring).

Allyl Isothiocyanate (Mustard Oil)		Allicin
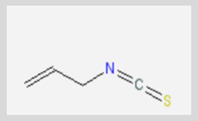	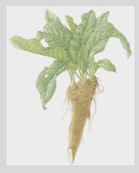	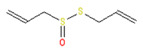 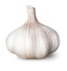
Horseradish (*Armoracia rusticana*)		Garlic (*Allium sativum*)
Methyl salicylate	Eugenol	Gingerol
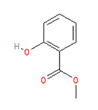	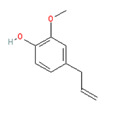	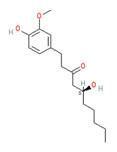
Wintergreen (*Gaultheria procumbens*)	Cloves (*Eugenia caryophyllata*)	Ginger (*Zingaber officianale*)
Nicotine	Thymol	Cynnamaldehyde
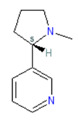	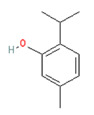	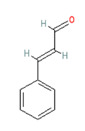
Tobacco (*Nicotiana* spp.)	Thyme (*Thymus vulgaris*)	Cinnamon (*Cynnamomum* spp.)

In reviewing anthropogenic/industrial TRPA1 agonists (Table 6, below), one is struck by the profusion of relatively small (and chemically reactive) molecules. Among others, these include surface disinfectants, chemical intermediaries, and combustion products. Equally striking is the fact that two of the compounds—methyl isocyanate (MIC) and methyl isothiocyanate (MITC)—differ by only one atom (the latter substituting sulfur for an oxygen atom). For readers steeped in the history of industrial toxicology, these two acronyms evoke images of multiple civilian casualties after the 1984 Bhopal disaster (MIC), and of thousands of dead fish (prized rainbow trout) floating down the Sacramento River after a 1991 train derailment releasing the soil biocide, Metam Sodium (breaking down into MITC). Less well-known is the fact that, in the latter incident (and for the first time in the published literature), persistent asthma-like symptoms were documented in a “bystander” (non-occupationally exposed) population after an area-wide airborne irritant exposure [44,45,46,47].

**Table 6:** TRPA1 Ligands/Reactants (Anthropogenic/Industrial).

Methyl Isocyanate	Methyl Isothiocyanate	Acrolein
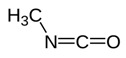	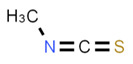	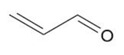
Industrial reactant (*pesticide precursor*)	Pesticide breakdown product (*hydrolysis of Metam Sodium*)	Combustion product *(e.g., cigarette smoke)*
Formaldehyde	Hypochlorite salts	Chloramine-T
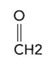	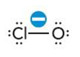	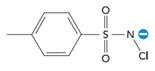
Surface disinfectant	Surface disinfectant	Surface disinfectant
Hydrogen peroxide	Hydroxyl radical	Ozone
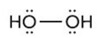	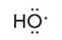	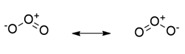
Disinfectant	Reaction intermediate	Air pollutant

### 2.3. Psychophysics

#### 2.3.1. Definitions and Methods

Psychophysics is defined as “the scientific study of the relation between stimulus and sensation” [48]. The term was introduced into the scientific literature by Gustaf Theodor Fechner in an 1860 publication, *Elemente der Psychophysik* (Elements of Psychophysics), building upon work he conducted with his mentor, Ernst Heinrich Weber. Perceptual tasks described and/or modeled in psychophysics include qualitative (description or naming of stimuli; discrimination between different stimuli) and quantitative (threshold detection of—or superthreshold intensity rating of—stimuli). The principles of psychophysical testing transcend the specific senses, and have their analogs in vision, hearing, touch, smell, taste, and chemesthesis.

Aggregations of psychophysical data may summarize individual nominal data (e.g., the likelihood of correctly detecting a stimulus at a given physical concentration or intensity), or scalar data (e.g., the ordinal rating of stimulus strength using a Likert-type scale or continuous rating of stimulus strength using a visual analog scale). An underlying principle of psychophysics is that the perceived strength of a stimulus changes according to the logarithm of its physical intensity. This principle harmonizes with the observation that “just noticeable differences” in stimulus strength are elicited by constant ratio change vs. a prior stimulus. Together, these principles have been termed the “Weber-Fechner Law” [48].

#### 2.3.2. Relationship of Nasal Chemesthesis to Olfaction

In everyday language, it would not be unusual for an individual to state that they had perceived an “irritating odor”. This statement makes sense even though the information being conveyed was derived from two different cranial nerves (CrN I for odor and CrN V for irritation). Since the apparent locus of sensation for both senses is the nose, it is natural to compare and contrast nasal chemesthesis and olfaction. Although most vapors or gases are able to act as both odorants and irritants, substances’ relative potencies for these two sensory modalities can diverge dramatically [49].

In acknowledgment of this fact, Alarie coined the term “odor safety factor”, which he defined for airborne irritant chemicals as the Threshold Limit Value^®^ (a non-binding occupational exposure limit promulgated by the American Conference of Governmental Industrial Hygienists) divided by geometric mean of published odor detection thresholds [50]. An example of a compound with a high odor safety factor is hydrogen sulfide, whose odor detection threshold of approximately eight parts-per-billion was less than one thousandth of its [then]-TLV based on mucosal irritation (ten parts-per-million). Given the excellent odor warning qualities of H_2_S (and other so-called “reduced sulfur compounds”), they are frequently added to odorless “natural gas” as a warning agent.

Both olfaction and nasal irritation can lend themselves to threshold detection determination via the so-called “forced-choice” method (with some caveats). In short, this procedure involves presenting gradually increasing concentrations of an active stimulus, paired (serially) with inactive “blanks” in random order, with the subject being asked to indicate which of each pair (or trio) was the “real” stimulus. While this method works well for generating odor thresholds, for irritant thresholds there is often a complication. If a given substance evokes odor at a lower concentration than irritation, one cannot avoid smelling the substance at a relatively low concentration prior to irritant detection and potentially confusing an odor threshold for an irritant threshold.

**Figure 7:**
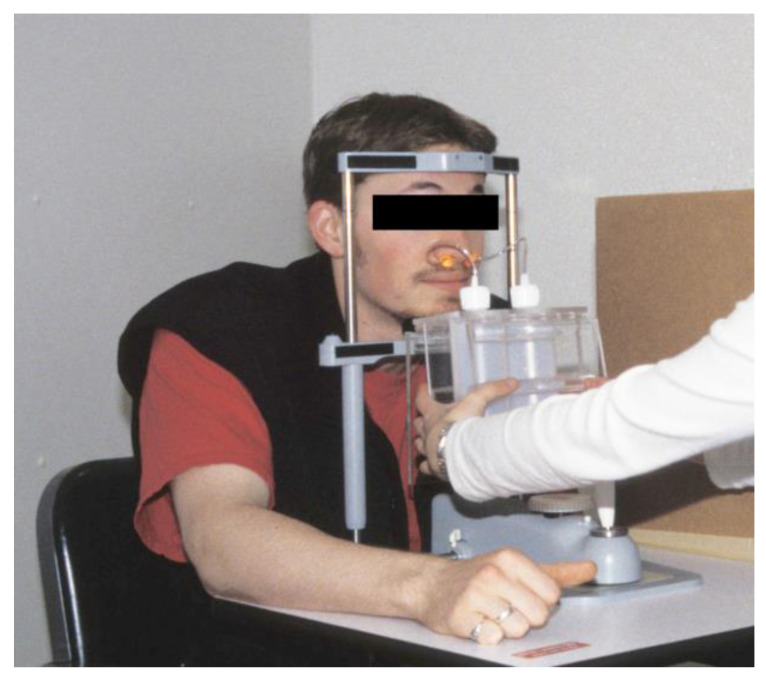
Procedure for obtaining human trigeminal irritation thresholds by lateralization. Active stimulus and “control” were presented with laterality randomized. Both active stimulus and diluent are colorless and transparent, thereby avoiding cueing. Source: Author.

Four approaches have been devised to avoid this pitfall: (1) First, testing can be limited to individuals who are anosmic (i.e., who lack the sense of smell). Although their detection thresholds may be biased upwards compared to normosmics, anosmics can only distinguish an irritant gas vs. clean air via its irritancy [51]. (2) Testing can employ a test compound that is irritating but [essentially] odorless. In this context, carbon dioxide pulses (which produce stinging via CO_2′_s hydration in mucous membrane water) are alternated with clean air in a forced-choice protocol. (3) Eye irritation thresholds (which closely parallel nasal irritation) can be obtained in a manner that precludes odor cueing [52]. (4) Finally, a test can be used that exploits a difference in humans’ central processing of chemosensory stimuli (humans being able to localize unilateral nasal irritation but not unilateral odorant stimuli). Using this test, an ascending concentration series of stimuli (paired with clean air blanks) is presented simultaneously to the two nostrils, with laterality randomized (Figure 7). Once lateralization (or “localization”) accuracy reliably exceeds chance, a nasal irritation threshold can be said to have been reached [53,54].

Of note, psychophysics is not the only approach to documenting human nasal irritation. Objective measures exist, including—peripherally—the so-called “negative mucosal potential” (NMP, which is measured with an electrode placed on the nasal mucosa). The NMP is thought to represent a “summation” potential generated by excitable cells in the nasal mucosa during irritation [30]. In terms of central processes, both chemosensory event-related potentials (“CSERPs”) on electroencephalography—and metabolic changes imaged with functional MRI (fMRI)—can distinguish between primarily olfactory and trigeminal responses to chemical stimuli [30].

#### 2.3.3. Applications of Trigeminal Psychophysics

In everyday life, the sense of smell generates relatively common clinical complaints that might come to the attention of otolaryngolgists, allergists, or primary care providers. Quantitatively impaired olfaction (hyposmia or anosmia, as seen during the COVID-19 epidemic) and—less frequently—qualitatively impaired olfaction (parosmias [distorted sensation in the presence of an odorants] or phantosmias [subjective odors in the absence of stimuli]) are well-known to medical providers [55]. Not surprisingly, then, there are commercially available “smell test kits” that can be administered in clinical settings (by specialists or non-specialists) to help in the diagnosis and treatment of these patients [56,57].

For nasal trigeminal function, by contrast, individuals are more likely to be bothered by excess than by deficient sensation. Clinically, both trigeminal neuralgia and cluster headaches are diagnoses marked by spontaneous and excessive trigeminal activity (see Pathophysiology, Section 3.4). From an environmental science perspective, population variability in trigeminal sensitivity is important, and—of relevance—there exists a research literature examining such variability as a function of age, sex, allergy, and smoking status [58,59,60]. For basic scientists interested in response mechanisms, there are also numerous published studies on the temporal and spatial integration of stimuli, agonistic and antagonistic interactions, and structure–activity relationships for different classes of compounds [30,61,62,63]. Despite this range of interested parties, nasal trigeminal test kits for use outside of a laboratory setting have yet to be made commercially available.

#### 2.3.4. Cooling and Airflow Sensation

While the central processing of chemesthetic signals is beyond the scope of this review, it is important to acknowledge there are peripherally based sensory phenomena in which different modalities converge. As noted above, there is an overlap between chemesthetic and thermal messaging in the TRPM8 channel, which is responsive to both menthol and cool temperatures. Further, nasal “congestion” or “obstruction” (a sense of impaired nasal patency) can be symptomatically alleviated by the topical application of menthol-containing substances within the breathing zone. Paradoxically, this occurs with no demonstrated change in nasal airway resistance [64]. Given the subjective overlap between the thermal and chemesthetic stimuli, it is not surprising that attention has focused on [perceived] mucosal cooling as a possible mediator for perceiving nasal patency.

Several lines of evidence support the cooling hypothesis: (1) Application of local anesthetic to the mucosa of the nasal cavity elicits the perception of nasal obstruction [65]. (2) Individuals with impaired nasal trigeminal function (particularly menthol perception) report greater nasal obstruction symptoms [66]. (3) Finally, computational fluid dynamic (CFD) studies (comparing CT-based nasal airway models of patients with and without subjective nasal congestion) predict impaired mucosal cooling in the former group [67]. Consistent with this hypothesis, so-called “empty nose syndrome” (a paradoxical perception of nasal obstruction in individuals who have undergone inferior turbinate reduction surgery to relieve obstruction) occurs despite the fact that affected patients generally have very low objective nasal airway resistance [68].

### 2.4. Reflexes—Skeletal, Autonomic, and Axon

The term “reflex” has been defined as “An action that is performed without conscious thought as a response to a stimulus…” [69]. A familiar example involves the sudden stretching of the quadriceps muscles of the anterior thigh (produced by striking the patellar tendon with a reflex hammer), resulting in an immediate and forceful contraction (shortening) of the quadriceps muscle group (the so-called “knee jerk” reaction). Involuntary musculoskeletal responses are but the most visible examples of reflexes in human biology, with macro phenomena (e.g., swallowing and breathing), intermediate phenomena (vasoconstriction, bronchoconstriction) and micro phenomena (neuromodulation and biochemical signaling) being less obvious but more numerous. In the following paragraphs, we will review a spectrum of reflexes triggered by the stimulation of the trigeminal nerve in humans.

Macro scale—Skeletal muscle reflexes (including both stretch and “nocifensor” [or defensive] reflexes) are “central” in nature, involving both an afferent (proprioceptive or nociceptive) and an efferent (motor) limb. For both, the reflex connection is at the level of the spinal cord or brainstem nucleus [70]. For proprioceptive stimuli, a stretch reflex maintains the postural stability of the organism in the face of an unexpected perturbation, whereas for nociceptive stimuli, the nocifensor reflex produces withdrawal from exposure to a noxious stimulus.

In our present discussion of trigeminal function, a relevant postural reflex is the so-called “jaw jerk”, in which stretch receptors in the masseter muscles can trigger muscle contracting (“clenching” of the jaw) by their sudden stretching. For a trigeminal nocifensor reflex, on the other hand, we have the so-called “blink reflex”, in which mechanical (or chemical) stimulation of the eye (particularly of the cornea) causes immediate blinking, again functioning to protect the organism from injury. In contrast to the jaw jerk (in which both the afferent and efferent limbs are carried by CrN V), the blink reflex involves CrN V afferents and CrN VII efferents. In addition, the blink reflex is frequently accompanied by autonomic activity, inducing tear production. Both blinking and tearing act to terminate an offending exposure. Of note, both increased blink rate and decreased tear film stability (faster tear film breakup time) can be demonstrated experimentally in a polluted airspace [71].

Intermediate scale—Reflexes involving the contraction or relaxation of smooth muscles (in the walls of blood vessels or the lower airways) as well as glandular secretion (e.g., tearing, rhinorrhea, or bronchorrhea) can sometimes be monitored using straightforward methods. Thus, blood pressure measurement, spirometry (or, in the case of tears, simple observation) can document the “response” portion of a stimulus–response relationship. Simple instrumentation (such as the placement of pre-weighed filter paper discs in the nose for a specified period of time) can help objectify the measurement of secretions.

**Figure 8:**
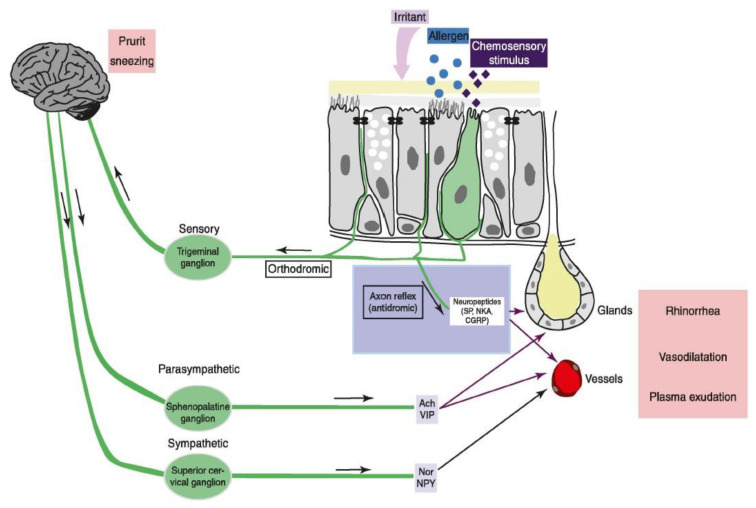
Innervation of (and reflex effects within) the trigeminal nerve distribution. n. Arrows indicated the direction of nerve impulse conduction (green) and biochemical diffusion (red). With permission from reference [21].

Pathophysiologically, these reflexes can involve multiple mechanisms, including centrally mediated autonomic (sympathetic/parasympathetic) activity and locally released neuropeptides as part of the axon or (“antidromic”) reflex (Figure 8). Of note, the so-called “non-adrenergic, non-cholinergic” (NANC) system actually encompasses three different response mechanisms, with specific peptides co-located with—and augmenting the effect of—the parasympathetic system (muscarinic acetylcholine + vasoactive intestinal peptide or VIP), the sympathetic system (adrenergic norepinephrine + neuropeptide Y or NPY) and the antidromic/axon reflex (substance P [SP], neurokinin A [NKA], and calcitonin gene-related peptide [CGRP]).

Of note, acute changes in airway caliber in the upper and lower airways involve only partially overlapping mechanisms. In the nose, vasodilation (i.e., engorgement of venous sinusoids) and plasma extravasation (tissue edema) are chief among acute response mechanisms. In the lower airway, while vascular mechanisms can be operative, acute smooth muscle contraction (“bronchospasm”) can also occur.

Micro scale—The same molecular messengers involved in reflex smooth muscle (or vascular) responses can also act as signaling (or modulation) agents in the nociception, chemotactic signaling, or modification of allergic processes (such as facilitating mast cell degranulation). While the neuromodulation of nociceptor function by endogenous compounds is beyond the scope of this review, it is reviewed in detail elsewhere [72].

#### 2.4.1. Respiratory Behavior (RD_50_)

A conspicuous group of reflexes manifested in response to inhalational exposures to irritant gases, vapors, or smokes are alterations in respiratory behavior. Depending upon the host and the stimulus involved, exposed organisms may exhibit coughing, sneezing, altered respiratory rate, changes in relative duration of inspiration vs. expiration, or frank breath-holding when presented with a noxious airborne substance. Based on known reflex changes to respiration (see Section 2.4.3, below), Alarie and colleagues—beginning in the 1960s—performed multiple experiments with rodents using a variety of airborne chemical agents with differing water solubilities and chemical reactivities. A number of response patterns emerged, yielding a chemical agent classification system of “sensory”, “pulmonary”, “bronchoconstrictor”, and “respiratory” irritants [73].

Sensory irritants were defined as agents whose major initial impact occurred in the mucous membranes of the eyes, nose or throat (i.e., trigeminally innervated structures) as a result of their high water solubility and/or chemical reactivity. They elicit frank burning or stinging sensations and are accompanied by the slowing of respirations (often including a pause during expiration). The involvement of the trigeminal nerve with this respiratory slowing was verified experimentally when rats either: (1) underwent direct electrical stimulation of their trigeminal nerve (resulting in slowed respirations) or (2) had their trigeminal nerve transected prior to exposure to a sensory irritant (in which case spontaneous respirations continued largely uninterrupted) [74].

Broncho-constrictors are agents—many of them also sensory irritants—that are capable of inducing acute airway narrowing in susceptible individuals (e.g., asthmatics). Affected individuals typically feel irritation in the substernal area of the chest as well as chest “tightness”, and may describe expiratory wheezing. Pulmonary irritants were defined as agents that—by virtue of their lack of warning properties—are able to penetrate into the deep lung and interfere with normal gas exchange. They elicit a sense of breathlessness without frank irritation, and are characterized by rapid, shallow breathing. Most broadly, the term respiratory irritant is applied to any airborne gas, vapor, particulate (including fume) or mixed exposure (e.g., smoke) producing irritation at any level of the respiratory tract (including the contiguous mucous membranes of the eye).

Based on their observations of respiratory slowing due to upper airway irritation, Alarie’s group went on to standardize a quantitative index of sensory irritation—the “RD_50_”—defined as the concentration of an airborne pollutant resulting in a 50% slowing of respiration in a standardized rodent assay [75]. This index was cross-validated with subjectively reported human data and went on to be recognized as a potential basis for occupational standard-setting [76].

Perhaps inspired by this experimental work, Cain and colleagues documented irritant-related changes in respiratory behavior in human subjects, eventually coining the term “transient reflex apnea” to describe the “involuntary disruption of breathing” that may occur during nasal irritant exposure [77]. Utilizing pulsed carbon dioxide as an odorless irritant, thresholds for this response were documented to be elevated in smokers vs. non-smokers [78], a finding that was replicated in at least one subsequent study [79].

#### 2.4.2. Trigemino-Facial Reflexes

As noted above, skeletal muscle activation initiated by trigeminal stimulation may involve efferent nerve fibers from CrN V (jaw jerk) or CrN VII (blink reflex). Another class of nocifensor reflexes utilizing the trigemino-facial (CrN V → VII) connection includes the autonomically mediated trigemino-facial reflexes resulting in rhinorrhea and/or tearing, usually as a result of either cold air or chemesthetic stimulation of mucous membranes. The prototype example of the former (i.e., cold air stimulation) is so-called “skier’s nose”, whereas for the latter (chemesthesis) it is “gustatory rhinorrhea”.

Cold air rhinorrhea/“Skier’s nose”

Skier’s nose is a dramatic example of what is referred to more generically as “cold air rhinitis”, “cold air rhinopathy”, or “cold air rhinorrhea”. As a sport-related phenomenon, it first appeared in the published literature in 1991 in a survey of 90 patients at a ski resort medical clinic, 96% of whom reported some degree of cold-induced rhinorrhea. The investigator subsequently conducted a double-blinded, placebo-controlled, crossover sub-study of a group of ski patrollers supplied with nasal sprays containing either atropine (a cholinergic blocker) or simple vehicle and found that all but one of them reported improvement when taking the active spray [80]. Ten years later, a somewhat larger (n = 144) sample of skiers was surveyed, albeit with a lower overall prevalence of cold-related nasal symptoms (~48%). A subset of subjects subsequently underwent blinded, placebo-controlled, crossover treatment with ipratropium bromide (IB) nasal spray (also a cholinergic blocker) prior to skiing, and an 80% treatment-related decrease was observed in self-rated cold-induced rhinorrhea [81]. Thus, in both studies, a parasympathetic reflex appeared to account for cold air rhinorrhea.

Gustatory rhinorrhea

Gustatory rhinitis (alternatively “gustatory rhinopathy” or “gustatory rhinorrhea”) refers to the phenomenon of copious watery rhinorrhea occurring while eating, particularly when the individual is consuming spicy food. The largest cross-sectional study of the phenomenon included nearly 600 respondents between the ages of 5 and 76 years, and in it both current or prior allergic rhinitis and smoking were associated with the phenomenon. On the other hand, the condition showed no apparent connection with gender, history of food allergies, asthma, or atopic dermatitis [82]. Comorbidity with other sensory (i.e., smell or taste) disorders has also apparently not been reported. In terms of mechanism, two studies have examined the efficacy of pre-treatment with a cholinergic blocker (either atropine or IB) applied topically to the nasal epithelium.

The first of these examined both symptom reporting and nasal lavage biomarkers after both control (e.g., crackers or pretzels) and irritant (chili peppers or horseradish) ingestion and did so on a repetitive basis after placebo [saline] and active [atropine] pre-treatment [83]. Among the 12 subjects studied, investigators found that atropine pre-treatment not only reduced the severity of symptoms, but also prevented the increase in both albumin and total protein content of nasal lavage fluid that was seen after irritant provocation after placebo pre-treatment. Further, the ratio of albumin to total protein (which would be expected to increase in the presence of plasma extravasation) was not altered in any of the experimental conditions, implying that vascular leak was not a contributing factor. Finally, the histamine content of the lavage fluid remained low under all experimental conditions, effectively eliminating mast cell degranulation as a mechanism of action. Per the authors, “food-induced secretions corresponded closely to the secretory pattern of cholinergically induced secretions”. Finally, the unilateral application of atropine to the nasal mucosa interrupted the secretory response unilaterally, but not bilaterally.

A second study included more experimental subjects than the previous study (n = 43) and utilized IB rather than atropine as the active treatment. In contrast to the prior study’s nasal lavage biomarkers, this study focused on the specific symptoms that subjects reported before and after irritant provocation. The study found an 8-fold increase in the VAS rating of “runny nose” post-irritant exposure (which was nearly completely ablated after IB pre-treatment) [84].

Together, the above two mechanistic studies imply that gustatory rhinitis is a centrally mediated parasympathetic (i.e., muscarinic) response, selectively interruptible by anticholinergic blockade. Both pharmacologic (IB) and surgical (posterior nasal nerve or vidian nerve neurectomy) interventions have been advocated [85].

Predating by a half-decade the substantial public interest generated by “skier’s nose” and “gustatory rhinitis” were several mechanistic studies of cold dry air (CDA-)-related nasal responses in human subjects. In addition to eliciting symptoms, the earlier studies examined biochemical endpoints (markers of glandular secretion, and vascular leakage) and contrasted the results with protocols involving immunologic triggering [86,87]. During this period, investigators also established that both early- and late-phase reactions can occur post-CDA challenge [88]. By the mid-1990s, academic researchers further established that unilateral CDA challenge elicits a bilateral secretory response, thereby supporting the model that a central (i.e., parasympathetic) response mechanism was at play [89,90]. As noted in the upcoming passage on nasal hyper-reactivity (Section 3.1, below), cold air provocation continues to be utilized as an index of non-specific nasal reactivity.

#### 2.4.3. Trigemino-Vagal Reflexes

Trigemino-facial reflexes are not the only examples of mixed central reflexes involving the nose as the sensory organ. Both the vagus (CrN X) and phrenic nerves can serve in the effector limb for trigeminally stimulated reflexes, with endpoints including both cardiovascular (see below) and respiratory (see both above and below) responses.

Cardiovascular (bradycardia)

The cardiovascular effects of nasally inhaled irritants were first documented in print in the late 19th century by Kratschmer [91]. In his description of the cardiac and pulmonary response in unanesthetized rabbits, Kratschmer described a simultaneous pause in heartbeat and respiration upon sudden exposure of the upper airway to an irritant gas, vapor, or smoke. In the case of respiration, inspiratory pauses as long as 15–20 s were not unusual. For the heartbeat, cardiac slowing (and decreased blood pressure) occurred for an equivalent time. In the remainder of his work, he carefully isolated exposures to different portions of the respiratory tract and concluded that—although isolated laryngeal exposures were not without effect—they did not reproduce the same dramatic effect as did the exposure of the nasal mucosa. Additionally, Kratschmer examined the effect of trigeminal or vagal transection prior to irritant provocation and found that bradycardia no longer occurred if either nerve were severed, but respiratory disruption remained if the trigeminus was intact but the vagus was interrupted. Since the vagus nerve can produce prominent cholinergic effects (including bradycardia responsive to atropine), it is not unreasonable to interpret the cardiac response to nasal irritation as a trigemino-vagal reflex, and the pause in respiration as instead involving the phrenic nerve (see also Section 2.4.1, above).

Respiratory (laryngospasm and bronchospasm)

Not content with simply explaining nasal irritation’s effects on cardiac function and thoracic respiratory behavior, Kratschmer also examined that stimulus’ influence on the glottic apparatus. He noted that mechanical irritation of the nose induced prompt laryngospasm in rabbits, not unlike what occurs with direct mechanical irritation of the vocal folds. Given that the glottis enjoys both afferent and efferent vagal innervation (through the superior and recurrent laryngeal nerves, respectively), it would seem that it may have redundant protective reflexes (i.e., both trigemino-vagal and vago-vagal). This is of particular contemporary interest, since so-called “paradoxical vocal fold motion” or PVFM (sometimes referred to as “vocal cord dysfunction” or VCD) is increasingly being recognized as cause for episodic dyspnea, with cases frequently being misdiagnosed as—or underdiagnosed in the presence of—asthma [92,93,94].

Changes in lower respiratory tract mechanics (e.g., bronchospasm) in response to nasal stimulation have been considered in the literature under the rubric of “nasobronchial reflexes”. Such reflexes have considered not only physical stimuli (such as cold air), but also chemical irritants, biochemicals involved in the allergic response (e.g., histamine), and antigens (in allergically sensitized individuals), and have been studied in both humans and experimental animals. Based on the volume of published studies, we will consider only physical stimulation (cold air) here, although the presence or absence of pre-existing asthma turns out to be an important covariate in these studies.

Cold provocation—whether directly applied to the nasal mucosa or to the skin of the face (also trigeminally innervated)—appears to be a dependable stimulus for increasing total airway resistance. Depending upon the specific study, this response can be seen in both asthmatics and in normal individuals, although the response is more robust in the former group [95,96,97,98]. The efficacy of combining nasal anesthesia and topical anticholinergic blockade to interrupt the cold air effect is not a universal finding, introducing some controversy [99]. Nevertheless, the preservation of the response in laryngectomized patients—as well as animal experiments in which either trigeminal or vagal section prevented the effect—continues to be consistent with a nasobronchial reflex mechanism [100,101,102].

## 3. Pathophysiology

### 3.1. Nasal Hyperreactivity

When considering the upper airway, a number of symptoms can be manifested. These include [perceived] nasal airflow obstruction (i.e., “congestion”), nasal hypersecretion (“runny nose”), post-nasal drip, sinusitis-related pain patterns, olfactory impairment, and nasal pruritus and sneezing. For all of these, the etiology can be allergic, nonallergic, or a combination of the two. For most of the nonallergic physiologic responses we have considered above as “reflexes”, a continuum exists between what one might term “normal” and “pathological”, since many of them have a [nominally] protective function. The dividing line is subjective, and depends upon the answers a number of questions: “How many symptoms?” “How frequently do they occur?” “How troublesome are they?” If these symptoms are prominent, frequent, troublesome, and have multiple triggers, then one might speak of “nasal hyperreactivity” (or NHR) on a clinical basis.

Typical symptom triggers reported by patients with NHR are nonspecific physical and chemical agents, and include exposures to cold air, spicy foods, smoke (particularly from tobacco), vapors from cleaning products, strong odors, exercise, alcohol, bright lights, and (in some cases) strong emotions. Although none of these are “allergic” triggers per se, they nevertheless affect at least 40% of individuals with allergic rhinitis and are a defining characteristic of a subcategory of nonallergic rhinitis sometimes referred to as “non-infectious”, “nonallergic rhinitis”, “vasomotor rhinitis” or “idiopathic rhinitis” [103,104]. Since the diagnostic criteria for this latter condition include both: (1) a lack of demonstrated allergic sensitization (i.e., negative epicutaneous skin tests, circulating antigen-specific IgE, or response to nasal antigen provocation) and (2) a paucity of inflammatory cells upon cytologic examination of the nasal mucosa, it has been proposed to label this condition as “nonallergic *rhinopathy*” rather than “nonallergic rhinitis” [105].

Aside from the clinical history, the most commonly employed diagnostic test for NHR is cold air provocation, utilizing either weight-of-secretions or objective nasal obstruction as endpoints [106,107,108,109]. The likelihood that NHR reflects neural hyperresponsiveness is supported by the fact that: (1) intranasal application of capsaicin (which is known to desensitize and/or ablate peptide-containing c-fibers) diminishes symptoms; (2) intranasal instillation of azelastine (a combined antihistamine and anti-inflammatory) reduces both nasal hyperreactivity symptoms *and* density of TRPV1 receptors in the nasal mucosa; and (3) subjects with “idiopathic rhinitis” and NHR have a lower threshold for perceiving and responding to irritants, including allyl isothiocyanate (mustard oil—a TRPA1 ligand) on objective tests of nasal irritation [110]. Given its considerable clinical relevance, further research into the pathophysiology and appropriate treatment of NHR is ongoing.

### 3.2. Headaches: Trigemino-Vascular Mechanisms

Chemically triggered headaches are a common complaint in industrial settings [111,112]. Although historically chemical headache triggers have been identified with direct vasoactivity, indirect mechanisms are also possible. An often-overlooked feature of trigeminal anatomy is the fact that CrN V—in addition to innervating the nasal mucosa, sinuses, facial skin, oral cavity and cornea/conjunctiva—also innervates the majority of the intracranial dura matter (Figure 2). Because of this shared innervation, the possibility exists that cross-talk (or “referred pain”) could occur within the CrN V distribution.

Exploring this possibility (using a rodent system), experimenters found: (1) The application of TRPV1 or TRPA1 agonists (capsaicin, mustard oil, or acrolein) to the nasal mucosa of rats resulted in increased blood flow through the animals’ middle meningeal artery; and (2) the pre-application of a CGRP antagonist to the meninges blocks this response [113]. Since CGRP release (resulting in vasodilation) could occur in meningeal nerve endings through either: (a) antidromal stimulation of shared nerve fibers or (b) cross-talk at the CrN V ganglion level, the investigators further explored the potential connection by double retrograde labeling nerve fibers serving the nasal mucosa and meninges. Interestingly, although they did not find any cell bodies in the CrN V sensory ganglion that were double-stained, they did find that the two sets of cell bodies were [spatially] closely associated with one another within the ganglion [114]. Finally, investigators also found that subacute low-dose irritant (acrolein) exposure potentiated the blood flow response to subsequent capsaicin and mustard oil exposure [115]. Given the prominence of vascular responses in current models of headache genesis, the physiologic and therapeutic aspects of potential trigemino-vascular mechanisms will likely continue to be the subject of active research.

### 3.3. Sinusitis: Solitary Chemoreceptor Cells, Taste Receptors and Trigeminal Activation

**Figure 9:**
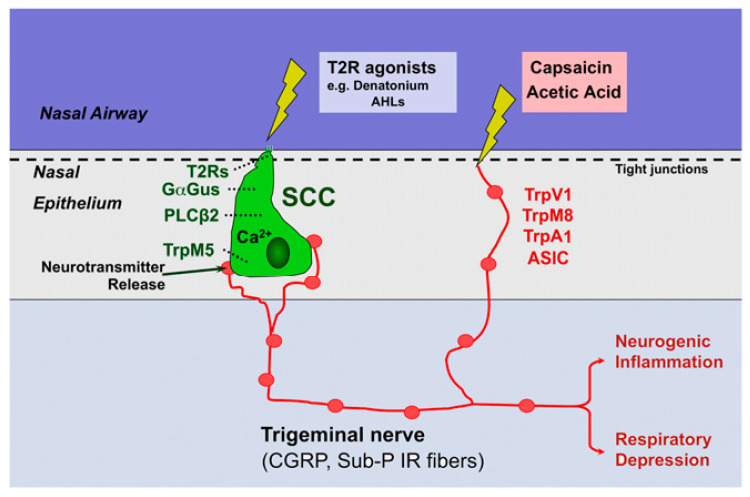
Parallel excitation of trigeminal nerve fibers by free nerve endings (invested with TRP nociceptors) and solitary chemosensory cells or “SCCs” (invested with bitter taste receptors). SCCs make direct contact with the airway lumen and respond to bitter substances that cannot diffuse beyond tight junctions to reach free nerve endings. With permission from reference [120].

When the TRPV1 ion channel was first cloned in 1997, the presence of specialized trigeminal-associated receptor cells (e.g., solitary chemosensory cells or SCCs) had yet to be convincingly documented in mammals. We have since learned that SCCs (and related “brush cells” and “tufted cells”) are not only present in human airways, but they express bitter taste receptors (T2Rs), along with the downstream apparatus that is normally associated with the sense of taste (e.g., TRPM5; α-gusducin). This class of cells can be stimulated by bitter substances in the airway, including quorum-sensing molecules related to developing bacterial biofilms [116,117,118]. Of note, the apical end of SCCs makes contact with the airway lumen, and hence SCCs can sense substances that cannot pass the tight junctions that shield trigeminal free nerve endings from contact. The human airway cell types in which this mechanism was first confirmed (ciliated epithelial cells) are both ubiquitous and capable of autonomous response since they contain motile cilia, whose beating is stimulated by their own cell’s activation as well as by lumenal nitric oxide (NO) production [119]. SCCs’ location—adjacent to trigeminal nerve endings—enables them to secondarily stimulate nearby trigeminal nerve endings (via cholinergic messaging) and thereby trigger the events characterizing neurogenic inflammation in parallel with TRP channel-equipped free nerve endings (Figure 9) [120,121].

The above sequence has profound clinical implications, since alterations in the taste pathway could potentially interfere with innate immune functions essential for the prevention of bacterial sinusitis. In actual fact, variants in the T2R38 gene (encoding for the receptor that recognizes phenylthiocarbamide [PTC] and propylthioluracil [PROP] as bitter substances) correlate with sinusitis susceptibility (as well as the likely necessity of sinus surgery) [122]. Of note, PROP has been used for many years as a standard test stimulus, differentiating “tasters”, “non-tasters”, and “supertasters” of bitter (and other) tastants [123]. Effectively speaking, then, a well-validated psychophysical test can now serve “double duty” as a marker of sinusitis susceptibility.

### 3.4. Intrinsic Rhinologic, Odontogenic and Neurologic Conditions Affecting Trigeminal Function

Most of the symptoms we have discussed up until this point have involved reflex responses to external stimuli. However, as previously noted (Section 2.3—Psychophysics), there are a number of intrinsic (rhinologic, odontogenic, and neurologic) conditions in which trigeminal sensations are generated internally. For some of these conditions (e.g., apical root abscesses or paranasal sinusitis), the connection between the inflammatory event and pain can seem intuitive. For others, a more occult (typically neurologic) source and mechanism is implicated. In the following paragraphs, we will briefly review these clinical entities.

In terms of intrinsic rhinologic conditions, acute and chronic sinusitis can produce pain patterns involving the scalp or face, often leading to a clinical diagnosis of “sinus headache”. This entity is acknowledged in the classification scheme of the International Headache society as “headache attributed to disorder of the nose or paranasal sinuses” [124]. Notwithstanding this recognition, the preponderance of the published literature suggests that the diagnosis of sinus headache is overused and frequently applied to individuals with more typical migraine headaches [124,125]. In so-called “mucosal contact headaches” (in which an anatomical protuberance—typically originating in either the nasal septum or a turbinate—forces its overlying mucosa to contact the [anatomically] opposite surface), the source of pain can seem more obvious. Before considering surgical correction, however, many clinicians, upon finding contact areas on physical examination and/or imaging, look for verification from a finding of temporary relief of discomfort after the topical application of a local anesthetic to the area of contact [126,127].

Neurologically, in addition to the relatively common entity of migraine headaches (characterized by episodic, pulsating cephalgia, unilateral or bilateral, with or without accompanying nausea, photophobia, or preceding aura), other intrinsic neural disorders can produce significant cranio-facial symptoms. Typically, these symptoms include episodic unilateral facial pain, with or without ipsilateral autonomic symptoms. Depending upon the frequency, duration, associated symptoms, and response to interventions, these may be labeled with one of several diagnoses.

Trigeminal neuralgia (sometimes referred to as tic douloureux because of occasional facial muscle spasms), is considered to inflict one of the most severe forms of pain that humans can endure. Symptoms include sudden and severe electric shock-like pain in the trigeminal distribution, often described as a “lightning bolt” or “stabbing” sensation [128]. While the majority of cases are considered idiopathic, secondary causes include trigeminal nerve compression (due to vascular, neoplastic, or infectious processes), or as a secondary manifestation of multiple sclerosis. In contrast to the related conditions discussed below, pain is either the sole or predominant manifestation of trigeminal neuralgia. More continuous trigeminal symptoms (“trigeminal neuropathy”) have been reported with sphenoid sinusitis and recurrent varicella-zoster infection (shingles), among other conditions [129,130].

On a related note, several diagnoses are grouped under the rubric of “trigeminal autonomic cephalalgias” (“TACs”), all of which pair unilateral trigeminal pain with autonomic symptoms (including some combination of rhinorrhea, nasal congestion, conjunctival injection, tearing, facial sweating, eyelid edema, ptosis, and pupillary changes). The most common of these is “cluster headache”, so-called because of the tendency of symptoms to cluster in time, with varying asymptomatic intervals between incidents. The condition is associated with cigarette smoking and male sex, may have a genetic contribution, and can be triggered by consumption of alcohol. Compared with the otherwise-similar diagnosis of “paraoxysmal hemicrania”, cluster headaches have a longer duration but decreased frequency of attacks, and the two conditions respond clinically to different interventions (oxygen and tryptans for cluster headache, vs. indomethacin for paroxysmal hemicrania). Further details on these and other, less frequently encountered TACs are described in two excellent review articles [131,132]. 

## 4. Therapeutic Considerations—Reflex Symptoms

Therapeutic interventions for trigeminally mediated reflex symptoms can involve agents targeting various steps in the response arc—from nociceptive transduction to central or peripheral reflex responsiveness to end-organ reactivity. Not surprisingly, comorbid conditions can be of importance since allergic inflammation can up-regulate multiple steps in this pathway. This “cross-talk” (between allergic and nonallergic processes) comes under the rubric of “neuromodulation”, a term we have used several times earlier in this text [72]. As a consequence of this phenomenon, an overlap exists in medications used for treating allergic and nonallergic conditions [133]. Put succinctly, the successful treatment of allergic rhinitis should, in principle, also reduce nonallergic reactivity.

This premise is supported by various lines of investigation. Laboratory provocation studies comparing allergic rhinitics vs. nonallergic subjects demonstrate lower trigeminal irritant thresholds (utilizing carbon dioxide or volatile organic compounds) and greater reflex reactivity (utilizing chlorine gas or acetic acid vapor) in the allergic rhinitic group [59,134,135]. Other psychophysical studies incorporating objective electrophysiologic measures (NMP and CSERPs) not only replicated these threshold differences but also found shorter electrophysiologic response latency times in the allergic rhinitics [60,136]. Although not validated in clinical intervention trials, these studies, in the aggregate, support the premise that the first step in ameliorating patients’ irritant-related symptoms is to ensure that their nasal allergies (if any) are properly diagnosed and treated.

Looked at sequentially, the starting point of the reflex arc (nociceptive transduction) constitutes a first potential therapeutic intervention point. Deliberate over-stimulation of TRP channels (particularly TRPV1) can desensitize [C-fiber] sensory neurons and can even ablate them. Consistent with this phenomenon, in multiple human studies, the local application of capsaicin to trigeminally innervated mucosae achieves symptomatic desensitization in a significant fraction of individuals [137,138,139,140,141,142]. Unfortunately, the treatment itself (causing local pain and necessitating multiple medical visits) can pose an acceptability issue, so future work in this area may substitute synthetic capsaicin analogs (potentially capable of desensitizing nerves without excessive discomfort) in place of native capsaicin.

Reflex responsiveness (either in CNS nuclei or as part of the axon reflex) is the next—and perhaps most difficult—step to study. On the one hand, we know that the subacute administration of a given irritant substance (e.g., acrolein gas) to experimental animals (rats) can sensitize them to subsequent provocation by different irritants [115]. On the other hand, alternating the administration of allyl isothiocyanate (mustard oil) and acetic acid vapor produces asymmetric desensitization effects [143]. Further, the fact that different protocols for repeated applications of irritants (e.g., mustard oil) can either sensitize or desensitize the human nose to subsequent exposures hints at a level of complexity that has yet to be successfully explained [144].

Focusing for a moment on end-organ reactivity, receptor blockers are a potential approach to managing symptoms for which upstream interventions are ineffective. Historically, a prime example is cholinergic blockade by ipratropium bromide (IB), which was approved in 1995 by the US Food and Drug Administration (FDA) for the treatment of nasal hypersecretion. As reviewed above, IB nasal spray is effective in reducing the symptoms of both cold air and gustatory rhinorrhea. Unfortunately, IB’s relatively short duration of action may have detracted from its widespread use for these indications. Although a successor agent has since been developed for treating chronic obstructive pulmonary disease (tiotripium bromide, with a longer half-life than IB), clinical trials have yet to be repeated for that agent in the context of rhinologic indications. Besides IB, other FDA-approved drugs for nonallergic rhinitis include local steroids (beclomethasone dipropionate and fluticasone propionate), azelastine nasal spray, as well as a fixed-dose combination of azelastine and fluticasone. Exactly where and how these anti-inflammatories act in the reflex arc is, unfortunately, far from clear.

More recently, attention has begun to be paid to TRP channel blockers in the prevention of neurogenic upper respiratory tract symptoms. So far, this approach has not lived up to expectations. Four studies were conducted with the pharmaceutical SB-705-498, which showed good patient tolerability and safety profiles. Of these studies, two involved seasonal allergic rhinitics (neither of which showed significant symptomatic improvement) [145,146]. The remaining two—both targeting nonallergic rhinitics—had mixed results: The first showed improved symptomatic tolerance to capsaicin challenge, but the second did not protect from a more “real-world” hazard: cold-dry air challenge-induced symptoms [147,148]. Overall, the investigators felt that the TRPV1-related pathway was contributory but not essential to nasal hyperreactivity (i.e., that there was “redundancy in symptom pathways”). Given a variety of nociceptive ion channels, this approach is likely to generate future research activity. For safety and efficacy data on investigational drugs, the reader is referred to the appropriate pharmaceutical literature.

Finally (and also under the category of therapy), two non-pharmacologic approaches have been applied to rhinitis symptoms. The first involved the nasal application of carbon dioxide to patients with either seasonal or perennial allergic rhinitis. Compared with the placebo (clean air) treatment, active treatment resulted in short-term decreases in total nasal symptom scores [149,150]. The second non-pharmacologic study utilized “kinetic oscillation” (50 Hz vibration via intra-nasal balloon) on patients with nonallergic rhinitis. Likewise, in the short term, the authors reported a significant (1-point on a 3-point scale) improvement in self-rated nasal stuffiness [151]. The ultimate fate of non-pharmacologic approaches remains to be seen, although the potential value of simple mechanical cleansing of the nasal mucosa (i.e., with saline nasal lavages) should not be overlooked.

## 5. Summary and Conclusions

The human trigeminal nerve is extraordinarily complex in both structure and function. Its sensory territory includes the surface of the face and anterior scalp, intracranial dura matter, cornea and conjunctiva, nasal passages, paranasal sinuses, and a substantial portion of the pharynx and oral cavity. Its sensory fibers provide information on joint and muscle position, temperature, pH, and touch (spanning from light touch to pain). Its range of nociceptive chemoreceptors is broad, and in tandem with the senses of smell and taste, provides a nuanced picture of the extracellular chemical environment encountered in the upper aero-digestive tract. Finally, its effector functions (the majority of which are reflex in nature) exploit skeletal muscle, autonomic, and local (axon and cellular) responses, in the process coordinating with other cranial nerves. The result is a “traffic control system” that is unmatched in complexity, enabling us to breathe, eat, and talk through a shared pharyngeal passage; enhancing our quality of life through spices and condiments; helping to protect us from toxic gases, vapors, and smokes; and combatting pathogenic organisms that would seek to use our paranasal sinuses as bases of operation.

From the perspective of the respiratory system, the vast number of studies dedicated to trigeminally related phenomena have paid off handsomely in terms of scientific and clinical insight, ability to translate knowledge into practical measures, and a broad picture of the interrelationship between phenomena which, when viewed on the surface, seem unrelated. Notwithstanding this optimistic assessment of the state of trigeminal research, momentum should be sustained, and future directions should include harmonization of data from natural vs. anthropogenic (or industrial) chemical irritants [152,153], exploration of mechanisms of neuroplasticity at the afferent-efferent interface, and a continued willingness to learn from “natural products and folk medicine”.

## Data Availability

Not applicable.
